# Maternal mental health and child well-being in Nigeria: A systematic review

**DOI:** 10.1177/20551029211012199

**Published:** 2021-04-29

**Authors:** Dung Ezekiel Jidong, Nusrat Husain, Tarela J Ike, Maisha Murshed, Juliet Y Pwajok, Ayesha Roche, Haruna Karick, Zubairu K Dagona, Gloria S Karuri, Christopher Francis, Shadrack B Mwankon, Pam P Nyam

**Affiliations:** 1 Nottingham Trent University, UK; 2 University of Manchester, UK; 3 Teesside University, UK; 4 University of Jos, Nigeria; 5 Federal University Oye-Ekiti, Nigeria

**Keywords:** child wellbeing, maternal depression, mental health, Nigeria, psychological intervention

## Abstract

Maternal mental health distress has a disease burden of severe adverse effects for both mother and child. This review identified maternal mental health concerns, their impact on child growth and the current practice of maternal healthcare for both mothers and their children in Nigeria. The Population, phenomenon of Interest and Context (PICo) model was adopted to formulate the review strategy, and five databases were searched for published articles between 1999 and 2019. Databases include Scopus, PubMed, ProQuest, Applied Social Science Index and Abstracts and Web of Science. Boolean operators (AND/OR/NOT) helped to ensure rigorous use of search terms which include ‘maternal’, ‘pre/peri/postnatal’, ‘mental health’, ‘mental illness’, ‘disorders’, ‘intervention,’ ‘Nigeria’, ‘child’, ‘infant growth’, and ‘wellbeing’. Thirty-four studies met the inclusion criteria, and extracted data were qualitatively synthesised and analysed thematically. Five themes emerged. These include (i) marital difficulties, (ii) relationship status of the mother, (iii) child’s gender, (iv) mode of child delivery and (v) child growth and development. The review showed a significant paucity of literature on the impact of specific maternal mental health problems on child physical growth and cognitive development. We concluded that culturally appropriate and evidence-based psychological interventions for maternal mental health problems would benefit Nigerian indigenous mothers. Therefore, the study recommends randomised controlled trials that are culturally appropriate and cost-effective for distressed mothers with children.

## Introduction

In Nigeria, an estimated 20%–30% of the population suffers from mental problems (Suleiman, 2016). However, only one out of every five persons who suffer from mental health problems can access any care (Abdulmalik et al., 2019). Less than 3% of the Nigerian government health budget goes to the prevention and treatment of mental health problems, with most of the funds allocated for the operational cost of dilapidated mental health facilities (Anyebe et al., 2019; World Health Organisation, 2019). Current mental healthcare treatment options in Nigeria are scarce and expensive (Anyebe et al., 2019; Madhombiro et al., 2021), and about 75% of Nigerians depend on out-of-pocket payments for such services (Aregbeshola and Khan, 2020). Not only is the lack of public health resources a significant challenge to the public in Nigeria (Jidong and Sanger, 2018), but more challenging for women during their maternal pre-, peri- and post-natal periods. Consequently, maternal mental health is a significant health concern that carries the disease burden of serious adverse effects for both the mother and child.

A common maternal health problem is depression. Depression affects about 19%–25% of women in their maternal periods (Gelaye et al., 2016). The majority of those affected are women in low and middle-income countries, which is significantly higher than women residing in high-income countries (Gelaye et al., 2016). Indeed, it is challenging to generalise epidemiological data regarding the prevalence of maternal mental health problems. It is unevenly distributed across the world regions and thus, low and middle-income countries contribute the least data due to the paucity of research. Therefore, it could be argued that data collected in low and middle-income countries on the quality of care service-users received is often left unnoticed and perceived as poor quality with significant information gaps on the safety, disease prevention and evidence-based treatment.

Previous studies conducted in the West have associated depression in mothers with many adverse outcomes, including poorer psychological health, disturbances in neurocognitive and physical development for the mother, and the child of the depressed mother (Gelaye et al., 2016). However, in Nigeria, where factors such as poor child growth and under-nutrition are more prevalent (Jidong et al., 2021a), it may be that these additional factors contribute to the development and maintenance of maternal mental health problems and distortions in the child development (Adewuya et al., 2007). Common symptoms of depression such as poor concentration, sleep and appetite disturbances, low mood and loss of interest are factors that can significantly impact the mother’s capacity to fulfil the demands of her child’s emotional and physical needs. Consequently, a mother who suffers from a mental health problem may be less responsive to her child’s cues, as partly explained in attachment theory. Attachment theory describes a psychological system motivated by innate behaviour to seek support from others, particularly in times of need (Ainsworth, 1979; Bowlby, 1969; Yip et al., 2018). For example, children of depressed mothers or caregivers have higher tendency to display avoidant or disorganised forms of attachment styles compared to non-depressed mothers (Martins and Gaffan, 2000; Toth et al., 2009). Although these associations seem to produce modest results which may be related to other intercorrelated factors, such as marital conflicts and lack of social support; however, the theory suggests that maternal care received at early childhood influences the child’s later emotions, behaviours and wellbeing (Rosmalen et al., 2015; Yip et al., 2018).

To the best of the researchers’ knowledge, this is the first systematic literature review that explored maternal mental health and its impact on child wellbeing in Nigeria. Although one study reviewed intervention literature on maternal and child health in Nigeria between 1990 and 2014 with an attempt to generate local evidence for policy initiatives (Kana et al., 2015), however, their study was biomedically driven with physical health concerns and did not explore maternal mental health. Therefore, the present study will explore the literature on the risk factors for maternal mental health and its adverse effects on the mother and child wellbeing.

## Method

### Protocol and registration

The review protocol was registered and published in the International Prospective Register of Systematic Reviews (PROSPERO, CRD42019149392). To ensure rigour, the study followed the Preferred Reporting Items for Systematic Reviews and Meta-Analyses (PRISMA). These rigorous procedures placed the study as high standard in line with global best practice of systematic review (Sideri et al., 2018).

### Selection criteria

Inclusion: (i) studies which demonstrated maternal mental health and child development or wellbeing outcomes, (ii) studies that provided interventions in the form of therapies, educational programmes or health visitor interventions, (iii) studies including mothers with children, who are self-identified in the studies as Nigerians and (iv) qualitative or quantitative studies that measure pre-and post-intervention and non-intervention studies that address maternal mental health issues and child development in Nigeria.

Exclusion: (i) studies involving interventions that do not focus on maternal mental wellbeing, (ii) studies in which the population is non-Nigerian women and (iii) studies that only measure the outcomes of child development or wellbeing.

### Search strategy and data abstraction

The research strategy was formulated using the PICo model (population [Nigerian mother and child], the phenomenon of interest [maternal mental health and child wellbeing], Context [psychology and psychiatry]) (Stern et al., 2014). To ensure further rigour, the Boolean operators (AND/OR/NOT) were adopted for strategic search (Abdulla and Krishnamurthy, 2016). Terms such as ‘maternal’, ‘pre/peri/postnatal’, ‘postpartum’, ‘mental health’, ‘mental illness’, ‘disorders’, ‘intervention,’ ‘Nigeria’, ‘child’, ‘infant growth’, and ‘wellbeing’ were used to search five databases; PubMed, ProQuest, Applied Social Science Index and Abstracts (ASSIA) and the Web of Science for published studies from 1999 to 2019.

The search yielded 471 studies, of which were screened for duplicated and removed (*n* = 449). The titles and abstracts of the retrieved studies underwent preliminary assessments. Two independent reviewers screened studies for relevance and assessed against the inclusion/exclusion criteria. Discrepancies were resolved by discussions with the wider research team and were either accepted or rejected, as appropriate. A total of 34 eligible studies were selected for the systematic literature review. Of the 34 studies, 18 looked at maternal depression, 3 studies investigated anxiety disorder, 2 studies examined the prevalence of psychiatric conditions during the postnatal period, which found schizophrenia as most common, followed by depression, 3 studies conducted a psychological intervention and 4 studies investigated risk factors associated with maternal depression and anxiety. Only five of the studies looked at child growth outcome in relation to maternal mental health.

### Data synthesis

A conceptual framework of synthesis was adopted (Torraco, 2005). Each source was critically analysed and rigorously evaluated for major themes, strengths, weaknesses and critical gaps using the synthesis matrix. The synthesis was conducted in five stages (i) relevant literature search, (ii) identified key ideas and elements, (iii) organised the key ideas and elements and (iv) synthesised information and build a case for new research intervention. Furthermore, an ‘index card method’ was adopted to identify and organise key ideas and elements in stages (ii) and (iii) identified above. In stage (iv), re-arranged each pile or category of the organised ideas (a) which showed a logical flow of information (b) compared and contrasted emerging themes (c) critiqued primary findings and discussions. The synthesis allowed for common themes in the literature to be identified. Data were categorised using a thematic coding approach. The key themes selected for analysis were agreed upon through discussion with the wider research team based on their relatedness and frequency concerning the review topic.

The lack of comparable outcomes from the results and intervention studies informed the use of a qualitative syntheses of the findings using an inductive approach. The strength of this approach is that it provides scope to capture nuances and unexpected outcomes. Initial manual coding through each of the eligible studies was identified by one reviewer and reviewed by a second independent reviewer to account for selection bias; inconsistencies in interpretations were resolved through discussion.

### Risk of bias and quality assessment

This study employed the Critical Appraisal Skills Programme (CASP) (2017) to assess qualitative studies. The CASP checklists allowed all papers to be critically analysed under the same criteria to reduce the chances of bias. For other quantitative and qualitative papers, a QualSyst tool was adopted (Kmet et al., 2004). This is a standard quality assessment criterion used for evaluating primary research papers from a variety of fields. The risk of bias assessment was conducted independently by two evaluators, and disagreements were discussed to reach a consensus, after which a third assessor resolved disagreements amicably.

Overall, the studies’ quality was deemed high (*N* = 23) or moderate (*N* = 8) quality. The studies with a CASP score below 5 and similar ratings on QualSyst were graded as weak quality (*N* = 3) associated with bias or poor methodology reporting. Common reasons for the moderate quality of studies were lack of diagnosis, high dropout rate or incomplete data, inconsistent with previous literature findings, single centre recruitment. A common limitation overall was the lack of longitudinal data collection and small sample size.

## Results

**Figure 1.**
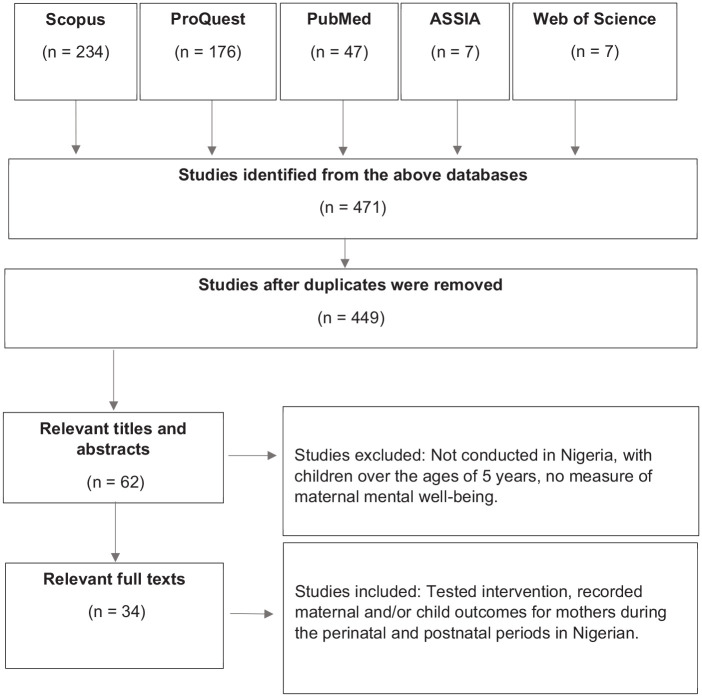
The trajectory of the literature review process and records.

Records received and the trajectory of selected studies from databases are illustrated in Figure 1 using the Preferred Reporting Items for Systematic Reviews and Meta-Analyses (PRISMA) (PRISMA, 2009). Subsequently, the characteristics of selected studies are also illustrated in Table 1.

**Table 1.** Characteristics of selected studies.

Author(s)	Aims	Sample population	Main findings	Conclusion	Limitations
Morhason-Bello et al. (2009)	Assess the effect of psychosocial support on labour outcomes.	585 recruited from the University College Hospital Ibadan.	Control 5 times likely to deliver by caesarean section (95% CI 1.98–12.05), higher pain scores (*p* = 0.0011).	Women with companionship had better labour outcomes compared to those without.	The response could suggest perceptions of quality of care, health workers’ attitude, ward environment.
			Experimental satisfying labour (OR = 3.3 95% CI = 2.15–5.04).		
Gureje et al. (2019)	Compare high-intensity treatment (HIT) with low-intensity treatment (LIT).	686 pregnant women who were registering for antenatal care were recruited.	HIT was effective for severe depression (OR = 2.29; 95% CI = 0.73, 1.01, 5.20; *p* = 0.047, higher exclusive breastfeeding.	Except for severely depressed perinatal women, no evidence to recommend HIT over LIT.	Enhanced care-as-usual was offered, rather than routine care as usual.
			Infant outcomes were similar across both interventions.		
Chinawa et al. (2016)	Prevalence of postnatal depression in Enugu.	Mothers attending postpartum clinics, 214 participants.	The prevalence of postpartum depression was 22.9%.	Prevalence in Enugu is comparable to African continents.	Not conducted in the community, a selection bias may be present.
Uwakwe (2003)	EPDS validity on Nigerian women.	225 women, Nnamdi Azikiwe University Teaching Hospital.	24 (10.7%) subjects had depression. An optimal cut-off score of 9, the EPDS had a sensitivity of 0.75 and specificity of 0.97.	EPDS distinguished depressed (*t* = 7.63, *p* < 0.001, *df* = 222).	Single site study.
Ndukuba et al. (2015)	Characteristics of those postpartum conditions.	76 women with postpartum psychiatric conditions.	Schizophrenia was commonest 48.7% 37/76, depression 22% 17/76 and mania 15% 11/76.	Encourage services into rural areas for early detection.	Referral bias.
Findley et al. (2013)	Results of maternal and child health programme.	2009 (*n* = 2129)–2011 (*n* = 2310) women with births in 5 years.	Anti-tetanus vaccination rates increased 69.0% to 85.0%.	The intervention was effective at changing infant care outcomes.	The assumption is under-reporting; a study push may present higher rates.
			Infant and child mortality declined greater in the intervention.		
Oyelohunnu et al. (2016)	Distress, mothers and child interaction.	98 women recruited from child immunisation clinic in Lagos.	An association was found between reduced maternal-child attachment interaction and maternal depression (*p* < 0.05).	Emotional disorders risk for reduced maternal-child interaction.	The design limits result in being generalised for causation.
Adewuya et al. (2008)	Postnatal depression on infant’ physical growth.	120 depressed and 122 non-depressed postpartum women.	Infants of depressed mothers’ significant poorer growth than non-depressed, and likely more episodes of diarrhoea.	Preventative methods of postnatal depression.	The study did not account for depression during pregnancy.
Ukpong et al. (2003)	Depression and preterm and full-term infants.	60 postnatal women, 33 preterm neonates, 27 full terms.	Mothers of preterm neonates (27.3%) statistically more depressed than mothers of full-term normal infants (3.7%).	These psychological difficulties are not usually detected.	93.9% preterm babies had various complications, impact wellbeing.
Sulyman et al. (2016)	The prevalence rate of postnatal depression, risk factors in Nigeria.	483 participants who delivered at a tertiary health institution.	Prevalence rate 22.4%. Risk factors unemployment (OR [OR] = 0.49, 95% CI = 0.27–0.86, *p* = 0.018), lack of support from husband (OR = 0.34, 95% CI = 0.19–0.60, *p* = 0.000).	The prevalence of postnatal depression was high and negatively impacts parent skills.	Single centred recruitment.
Onyemaechi et al. (2017)	Self-esteem, social support and age on postpartum depression.	116 mothers recruited from 5 hospitals presenting for postpartum check-ups.	Participants with low self-esteem had higher scores (*F* = 14.097, *p* < 0.0001). Subjects with low social support experienced higher rates of depression (*F* = 4.368, *p* < 0.05).	Social connection is an essential factor for mothers.	The sample size was moderate.
Adewuya et al. (2007)	Prevalence of the depressive disorder in late pregnancy.	180 women in late pregnancy (32 weeks and above) recruited from antenatal clinics.	Risk factors single (OR = 16.67, 95% CI = 3.17–87.76), divorces/separated (OR = 11.11, 95% CI = 1.55–19.65), polygamous (OR = 3.92, 95% CI = 0.94–16.66), lack of social support (OR = 6.08, 95% CI = 1.42–26.04).	Depression is common in late pregnancy in Nigerian women. Significant predictors are mainly social and family factors.	The sample size was moderate.
Thompson and Ajayi (2016)	Prevalence of antenatal depression and risk factors, in Nigeria.	314 women attending antenatal clinics.	The prevalence rate was 24.5% for antenatal depression.	Antenatal depression is prevalent in Nigerian women. Interventions will need to address risk factors.	The sample was screened for probable depression; no case of depression could be confirmed.
			Risk factors marital status (*p* = 0.010), gender-based abuse (*p* = 0.034), history of previous caesarean section (*p* = 0.032).		
Ayinde et al. (2018)	Quality of care received by women.	20 facility managers, 218 women recruited.	The perinatal women rated the service as good quality (96%) and satisfied with the care received (98%).	Major inadequacies in the maternal care facilities.	Limited awareness of quality care indicated by low service expectation.
Owoeye et al. (2006)	EPDS scores in a group of Nigerian women.	252 women were recruited from a 68-bed maternity hospital.	23% scores 12 and above on the EPDS. Risk factors marital conflict, rejected paternity of the new-born baby, (OR = 9.44, CI = 2.35–37.82) single status (OR = 5.76, CI = 1.96–16.94).	Postpartum depression is a major complication during childbirth in Nigeria.	The hospital-based study may not reflect the wider community.
Adewuya (2005)	Maternity blues in Nigerian postpartum women.	502 completed the screening questionnaires, giving a response rate was 86.3%.	Prevalence was 31.3%, symptoms peaked on the fifth day.	Differentiations across cultures for maternity blues must be considered in strategies for prevention.	There was a high rate of exclusion and drop out in the study.
			Significant predictors female baby (OR = 2.82, 95% CI = 1.53–4.12), and single mothers (OR = 3.35, 95% CI = 2.26–5.64).		
Adeponle et al. (2017)	How culture shapes social determinants and depression.	14 mothers with perinatal depression, 14 family caregivers, 11 health carers.	Perinatal depression associated with sociomoral concerns over gender roles. Risk factors, having a female child, ‘spiritual attack’ and not resting sufficiently after childbirth.	Assessments and interventions to consider local social contexts and meanings of depression.	Possible selection bias as women recruited only from hospitals, health centres, faith and traditional centres.
Bakare et al. (2014)	National Programme on Immunisation in southeast Nigeria	408 mothers recruited from 2 main University teaching hospitals in Enugu State.	15.2% met a diagnosis for depression.	The programme may provide evidence to support the early screening of mothers for depression in Nigeria.	The growth parameters of the infants were only studied once, and therefore no follow-up.
			Postnatal depression significantly associated with the growth of weight and length of infants, but not head circumference.		
Adewuya et al. (2006)	Anxiety disorders in late pregnancy.	181 women in late pregnancy (32 weeks and above).	The rate of anxiety disorder in pregnant was 39.0% compared with 16.3% in the non-pregnant (*p* < 0.001).	The rate of social anxiety was higher in pregnant than non-pregnant.	The onset of the disorder was not taken into account.
Agbaje et al. (2019)	Depression, anxiety.	270 postpartum women.	34.6% postpartum depression, 33.3% anxiety symptoms.	High prevalence in Nigerian women.	Considers one type of care service.
Ishola et al. (2018)	Postpartum depression among mothers of preterm babies in Lagos.	152 mothers recruited from the Massey Street Children’s Hospital.	Reliability (*α*) of 0.91, validity exploratory factor analysis cognitive, emotional difficulty (*α* = 0.92), hopelessness, suicidal ideation (*α* = 0.93), distress (*α* = 0.71).	Screening tool valid and easy to administer for postnatal depression mothers of preterm babies.	The study did not compare with women with full-term babies to ascertain its discriminant validity.
Ukpong (2011)	Psychological morbidity in mothers of preterm infants.	57 mothers of preterm infants were recruited.	36.8% high levels of psychological distress, 19.3% depression, 12.3% of cases were of anxiety. Psychological morbidity and depression related to neonatal birth weight.	Detection and management of postnatal psychological morbidity should be a priority in Nigeria.	The sample size was small, and the study was not longitudinal.
Loto et al. (2010)	Mode of delivery, self-esteem and parenting self-efficacy.	115 women had a caesarean section. 97 matched control of mothers had a vaginal delivery.	Self-esteem was lower for caesarean section (*p* = 0.006 and at 6 weeks (*p* < 0.001). Parent-child relationship scores lower caesarean (*p* < 0.001, OR = 4.71, 95% CI = 1.75–14.71).	Nigerian women who deliver via caesarean; low self-esteem, poor parenting self-efficacy in postnatal.	It did not account for mothers who had low self-esteem before delivery.
Odinka et al. (2018)	Postpartum anxiety and marital satisfaction.	309 nursing mothers were recruited.	Postpartum anxiety 31.1%, 33.3% depression. Co-morbid depression, anxiety (22%), high marital dissatisfaction.	Association marital dissatisfaction and depression and anxiety.	The design of the study limits the ability to draw causal conclusions.
Adefuye et al. (2008)	Postpartum mental disorders.	January 1988 and December 2007 were 9085 deliveries.	There were 27 cases of puerperal mental illness, which gave an incident rate of 2.9 per 1000 births.	The high incident rates of mental illness in Sagamu, Nigeria.	The findings were based on one location.
Abiodun (2006)	Postnatal depression in primary health care.	Women recruited 6 weeks postdelivery.	Prevalence 18.6%, depression predicted not having desired gendered baby (OR = 2.86; 95% CI = 1.62–5.93; *p* < 0.05).	EPDS should be incorporated into the routine screening.	Selection bias for those who are depressed and do not attend clinics.
Adewuya et al. (2005)	Post-traumatic stress disorder after childbirth.	876 women attending postnatal clinics at 6 weeks postpartum.	Prevalence for PTSD was 5.9%. Risk factor emergency caesarean section (OR = 7.31, 95% CI = 3.53–15.10).	PTSD is higher in Nigerian women than those in western cultures.	PTSD symptoms may have existed before childbirth.
Ukpong et al. (2003)	Psychological distress in the postpartum period.	33 preterm neonates and 27 mothers of full-term infants.	Mothers of preterm neonates significantly experienced more emotional distress and depression than full-term normal.	A multidisciplinary approach to address postnatal distress.	Neonatal health status could have affected maternal wellbeing.
Adewuya et al. (2005)	Sociodemographic and obstetric risk factors.	876 women recruited 6 weeks postpartum.	Depressed mothers statistically single mother (OR = 3.44, CI = 2.15–5.53), had emergency caesarean sections (OR = 3.58, CI = 1.72–7.48) and female babies (OR = 2.74, CI = 1.87–4.03).	Prevalence rates to be similar across cultures but identified risk factors to differ significantly.	A standard diagnostic instrument was not used to measure depression.
Lawal and Idemudia (2017)	Breastfeeding, self-efficacy, health locus of control on wellbeing.	291 breastfeeding mothers recruited Lagos state, Nigeria.	Breastfeeding self-efficacy influence sense of autonomy *F*(1, 283) = 31.183, *p* = 0.000, partial *η*^2^ = 0.099, positive relations with others *F*(1, 283) = 24.402, *p* = 0.000, partial *η*^2^ = 0.079, self-acceptance *F*(1, 283) = 37.043, *p* = 0.000, partial *η*^2^ = 0.116.	Breastfeeding mothers need to feel more confident in their breastfeeding abilities and feel they have control over their health.	The cross-sectional approach limit generalising the findings.
Fatoye et al. (2004)	Late pregnancy and matched controls for emotional distress.	156 pregnant women (gestational age 36 weeks or above) and 156 non-pregnant women.	Higher depression in pregnant (*M* = 33.58) than controls (*M* = 28.28) (*t* = 3.99, *df* = 310, *p* < 0.001). Higher mean anxiety (*M* = 39.29) than controls (*M* = 31.86) (*t* = 4.17, *df* = 310, *p* < 0.001). Polygamy, mode of previous delivery risk factors.	Women significantly had higher levels of anxiety and depression in late pregnancy compared to non-pregnant controls.	Participants were recruited from one health centre.
Ukpong and Owolabi (2006)	Postpartum emotional distress, caesarean section, vaginal delivery.	47 who had caesarean section and 47 matched controls who had a vaginal delivery.	The difference in mean scores for the index group 6.66 (6.37) was higher (*p* < 0.001) than the control group 1.23 (2.98). 42.5% of the women who has a caesarean significant emotional distress.	Caesarean childbirth may predispose Nigerian women to adverse psychological distress.	The data was self-reported, and therefore, response and sample biases may be present.
Tungchama et al. (2017)	Quality of Life of women with depression.	531 mothers 6–8 weeks postpartum in Nigeria.	Mothers had a significant poor perception of Quality of Life (QoL). Emergency caesarean significant (*B* = −11.89, *p* = 0.26).	The predictors of QoL indicated the outcome of postnatal depression in mothers.	Cross-sectional design limits the inference on the causality of the variables.
Adewuya et al. (2008)	Depression, postpartum and non-postpartum.	876 6 weeks postpartum, 900 matched non-postpartum.	Depression in 14.6% of postpartum and in 6.3% in the non-postpartum, significant (*t* = 8.919, *df* = 875, *p* < 0.001).	The prevalence of postnatal depression in Nigeria is comparable to Western.	Prevalence based on women who attended immunisation clinics.

### Themes

Five key themes emerged from the analysis of selected studies (i) marital difficulties, (ii) relationship status of the mother, (iii) child’s gender, (iv) mode of child delivery and (v) child growth and development. There was limited data obtained on the cognitive development of the child of a depressed mother. Most of the studies looked at maternal depression during the perinatal and postnatal periods.

## Discussion

This review harnessed relevant literature on maternal mental health and child’s healthy development in Nigeria. Risk factors such as socio-economic hardship and lack of access to adequate healthcare services showed adverse effects such as maternal distress and poor child’s physical and mental development. A chaotic family environment due to maternal mental health challenges affects the child’s ability to form a secure attachment (Bowlby, 1969; Parsons et al., 2012). More so, depressed mothers are emotionally withdrawn or display increased negative behaviours that reinforce an insecure environment for their children (Toth et al., 2009). Insecure or unreliable caregiving predicts lower levels of emotional regulation, negative self-perception, or others’ negative perceptions (Yip et al., 2018). The model demonstrates how factors such as marital tension or the caregiver’s distress could cause a child to experience problematic growth and development even when these tensions are relieved.

Marital difficulties lead to poor psychological adjustment during the perinatal and postpartum periods. Comorbidity of depression and anxiety had significantly higher marital dissatisfaction. For example, the strongest correlation between marital dissatisfaction with depression and anxiety is the phrase that “my partner doesn’t confide in me” (Odinka et al., 2018:6). Findings showed that women with postnatal depression were more likely to report receiving inadequate support from their husbands (Adefuye et al., 2008; Owoeye et al., 2006; Suleiman, 2016). A slightly different effort is expected of a new mother, such as learning to cope and adjust with the baby. This expectation is more challenging with postnatal depression. Therefore, accessible and cost-effective care is essential for the mother to manage emerging psychosocial stress and enable the child’s healthy development. For example, an aspect of the review correlated perinatal maternal depression and husbands not caring with a potential lack of trust and confidence in their spouse (Adeponle et al., 2017). However, the study did not acknowledge or record difficulties associated with pregnancy, contributing to psychological distress and its impact on marital disharmony (Odinka et al., 2018). Intriguingly, two studies showed postpartum schizo-affective disorders as a common mental health problem in Nigeria and related marital disharmony as a strong feature in maternal mental health problems (Adefuye et al., 2008; Odinka et al., 2018). The high incident rate of schizophrenia may be related to the poor diagnosis of other mood or affective disorders (Adefuye et al., 2008; Ndukuba et al., 2015; Odinka et al., 2018).

Indeed, a bidirectional argument could be made between maternal mental health problems and marital difficulties. Within the Nigerian context, the mother’s relationship status could be considered essential to their mental health and wellbeing. The review identified six studies that found prevalent of distress during pregnancy and in the postnatal periods for single mothers, divorced, separated or in polygamous marriages (Adewuya, 2005; Adewuya et al., 2007; Fatoye et al., 2004; Owoeye et al., 2006; Thompson and Ajayi, 2016; Ukpong and Owolabi, 2006). A slightly contrary view suggested that the mother’s status may be related to the lack of social support received compared to married women (Ukpong and Owolabi, 2006). Within the Nigerian context, conception and childbirth in marriage are highly reverends. There seem to be high levels of cultural stigma associated with the mother’s single status, which may contribute to a depressive mood. Such women may be viewed as promiscuous, and single parenting is often regarded as unacceptable in some traditional African cultures (Fatoye et al., 2004). More so, polygamous marriages are practised in Nigerian culture, and mothers from such families are likely to experience significant emotional distress due to less spousal support (Fatoye et al., 2004). However, there seems to be no identifiable causal link between mothers’ relationship status and depression in the cross-sectional research design of the reviewed studies.

Furthermore, having a female baby when a male baby was desired as a significant predictor for maternal depression (Abiodun, 2006; Adeponle et al., 2017; Adewuya, 2005; Adewuya et al., 2005). The Nigerian culture has a deep-rooted preference for male children, which might be due to their higher social status for potential inheritance and intergenerational retainment of the ‘family’s surname’ (Adeponle et al., 2017). Consequently, mothers could be blamed for the gender of their child, which could be threatening and, in some cases, may result in a marital break-up or the husband marrying another wife (Adewuya et al., 2005). It has also been suggested that a male child stabilises the mother’s marital status (Abiodun, 2006). This risk factor has been significant in other studies conducted in developing countries (Abiodun, 2006).

Child delivery via caesarean session is significantly associated with mental health problems in mothers. This is also consistent in Western and non-Western countries where often the procedure is unplanned or due to complications during child labour (Adewuya, 2005; Adewuya et al., 2005; Fatoye et al., 2004; Loto et al., 2010; Morhason-Bello et al., 2009; Thompson and Ajayi, 2016; Tungchama et al., 2017; Ukpong and Owolabi, 2006). However, there were no significant depressive scores between elective or emergency caesarean surgery, which suggests that the correlation could be related to the general fear of surgical procedures (Fatoye et al., 2004; Ukpong and Owolabi, 2006). However, in Nigerian culture, the caesarean section’s process could be perceived as a failure of womanhood (Ukpong and Owolabi, 2006). The procedure was also found to significantly correlate with lower self-esteem and parenting self-efficacy in women who could be related to societal expectations of child delivery without medical intervention (Loto et al., 2010).

Other studies examined child growth and development concerning maternal depression (Adewuya et al., 2005, 2008; Bakare et al., 2014; Findley et al., 2013; Ukpong et al., 2003). One study investigated the maternal and child outcomes in a community-based health programme (Findley et al., 2013). The research showed that at 6-months postnatal stage, the children of the depressed mothers had poor growth compared to the non-depressed mothers (Adewuya et al., 2008). Also, depressed mothers were more likely to engage in maladaptive caregiving behaviours such as disrupting breastfeeding. Therefore, the children of the depressed group had more diarrhoea episodes and other infectious diseases. Consequently, there were strong correlations between children of depressed mothers who had poor growth (height and weight) than children of non-depressed mothers (Adewuya et al., 2008). An earlier study associated the severity of initial neonatal illnesses due to depression in mothers and suggested that preterm children with various complications may have had the adverse impact from maternal distress (Ukpong et al., 2003).

This systematic review’s implicit findings suggest that inequality of gender is one of the most significant issues to maternal mental health in Nigeria. For example, maternal mortality caused by pregnancy-related problems are still relatively high in Nigeria and can be a crucial indicator of gender inequality (Matthew et al., 2020). Gender disparity may include a lack of women’s access to education, healthcare, social rights and high child marriages rates (United Nations International Children’s Fund, 2019). Child marriage alone cost the Nigerian economy a lost earnings estimate of USD 7.6 billion (World Bank, 2017). These factors of gender disparity have placed most Nigerian mothers in the position of low-income and socio-economic disadvantage (Edjumudo, 2013; Matthew et al., 2020). Women socio-economic empowerment is essential to closing the gender disparity gap (Matthew et al., 2020). Thus, skill-based intervention could be a valuable preventative strategy in developing and promoting sustainable maternal mental health in Nigeria.

Consequently, the review showed that mentally distressed mothers and caregivers could not fulfil their children’s physical and psychological needs. Therefore, the development of preventive measures and new interventions could draw initiatives and potential ideas from the current review. However, the present findings could be taken with caution due to their limitations. For example, most of the reviewed studies’ recruitment sites were public facilities, and none of the studies assessed symptoms that may have existed in the earlier stages of pregnancy. The research identified healthcare service in public health facilities as persistently of low quality in Nigeria, making private healthcare facilities unavoidable and leaving mothers vulnerable to ‘hospital induced’ depression, which is an under-researched risk factor (Thompson and Ajayi, 2016). Additionally, much of the data was obtained using a cross-sectional design, limiting the generalisability of the findings and implication of the causality of factors predicting maternal mental health issues.

One of this review’s objectives was to synthesise published intervention studies focusing on maternal mental health and child well-being in Nigeria and better understand the association between maternal mental health, child physical growth and cognitive development. The limitation is the lack of comparable data on child health outcomes and maternal mental health interventions in Nigeria. Findings revealed a limited or non-existence of sustainable evidence-based intervention for mothers identified with maternal mental health problems in Nigeria. Besides, there is little data on maternal mental health problems on the children’s long-term growth and cognitive-developmental in the country.

Although the factors associated with maternal mental health in the present study may not be significant in non-African settings. Therefore, cultural differences should be accounted for when formulating preventive measures and mental health interventions (Jidong et al., 2021b). Essentially, strategies may focus on improving the social network for mothers to address the trauma associated with childbirth, alongside supportive measures to further address possible or perceived lack of spousal or family support (Jidong et al., 2021a). Provision of culturally appropriate intervention models that account for common risk factors that are context-specific within a low- and middle-income region like Nigeria, which may allow for targeted and effective intervention. Based on the present review, it is recommended that cost-effective and evidence-based interventions proven successful in other countries could be culturally adapted, tested and revalidated for the Nigerian context in randomised controlled trials. Adaptation should consider the cultural appropriateness of the intervention materials and utilisation of local resources.

This study’s rigour and credibility are enshrined in how the systematic literature review is well-articulated. For example, PRISMA, PICo model, Boolean operators, the robust risk of bias assessments tools and the Conceptual Framework as the strategy for data syntheses of selected studies. Thus, the study’s originality and significance are also embedded in the paucity of literature concerning maternal mental health in Nigeria despite its disease burden of severe adverse effects for mentally distressed mothers and their children – threatens the intergenerational transmission of disease burden to these vulnerable population. Consequently, the current review could inform the development of preventive measures and new interventions for maternal distress and improves child wellbeing.

## Conclusion

The study systematically reviewed the literature on maternal mental health and child wellbeing in Nigeria. Maternal distress is a significant concern with a disease burden of severe adverse effects for both mother and child. The study explored and identified maternal health concerns, their potential impact on child growth and the current practice of maternal healthcare for both mothers and their children in Nigeria. The synthesised literature showed that future intervention strategies may improve the social network for mothers to address the trauma associated with childbirth, alongside supportive measures to further address possible or perceived lack of spousal or family support for mentally distressed mothers. The Nigerian cultural context and the low- and middle-income nature of the country were essential. Therefore, randomised controlled trials that are culturally appropriate and cost-effective for distressed mothers with children will be beneficial.
